# 13-[4,5-Bis(methyl­sulfan­yl)-1,3-dithiol-2-yl­idene]-6-oxa-3,9,12,14-tetra­thia­bicyclo­[9.3.0]tetra­dec-1(11)-ene

**DOI:** 10.1107/S1600536809037301

**Published:** 2009-09-26

**Authors:** Rui-Bin Hou, Bao Li, Tie Che, Bing-Zhu Yin, Li-Xin Wu

**Affiliations:** a Key Laboratory of Organism Functional Factors of Changbai Mountain, Yanbian University, Ministry of Education, Yanji 133002, People’s Republic of China; b State Key Laboratory of Supramolecular Structure and Materials, College of Chemistry, Jilin University, Changchun 130012, People’s Republic of China

## Abstract

In the title mol­ecule, C_14_H_18_OS_8_, one O atom, two S atoms and six C atoms form an 11-membered ring with a chair-like conformation; the planes of the two five-membered rings connected by a carbon–carbon double bond form a dihedral angle of 29.97 (11)°. In the crystal, pairs of weak inter­molecular C—H⋯S hydrogen bonds link two mol­ecules into inversion dimers.

## Related literature

For background to crown ether-annulated 1,3-dithiol-2-thio­nes, see: Hansen *et al.* (1993 ▶). For the synthesis, see: Chen *et al.* (2005 ▶). For a related structure, see: Hou *et al.* (2009 ▶)
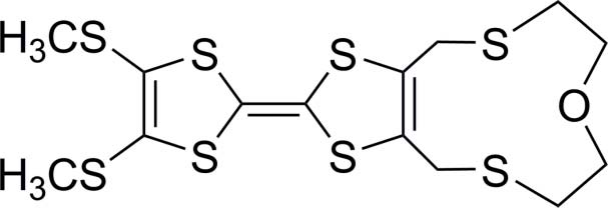


## Experimental

### 

#### Crystal data

C_14_H_18_OS_8_
*M* *_r_* = 458.76
Triclinic, 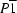

*a* = 8.4542 (17) Å
*b* = 10.158 (2) Å
*c* = 13.612 (3) Å
α = 105.00 (3)°
β = 97.83 (3)°
γ = 112.22 (3)°
*V* = 1008.8 (3) Å^3^
*Z* = 2
Mo *K*α radiation
μ = 0.88 mm^−1^
*T* = 291 K
0.14 × 0.12 × 0.12 mm

#### Data collection

Rigaku R-AXIS RAPID diffractometer
Absorption correction: multi-scan (*ABSCOR*; Higashi, 1995 ▶) *T*_min_ = 0.886, *T*_max_ = 0.901
9961 measured reflections
4572 independent reflections
3655 reflections with *I* > 2σ(*I*)
*R*_int_ = 0.032

#### Refinement

*R*[*F* ^2^ > 2σ(*F* ^2^)] = 0.056
*wR*(*F* ^2^) = 0.170
*S* = 1.10
4572 reflections
210 parameters
18 restraints
H-atom parameters constrained
Δρ_max_ = 1.09 e Å^−3^
Δρ_min_ = −0.64 e Å^−3^

### 

Data collection: *RAPID-AUTO* (Rigaku, 1998 ▶); cell refinement: *RAPID-AUTO*; data reduction: *CrystalStructure* (Rigaku/MSC, 2002 ▶); program(s) used to solve structure: *SHELXS97* (Sheldrick, 2008 ▶); program(s) used to refine structure: *SHELXL97* (Sheldrick, 2008 ▶); molecular graphics: *PLATON* (Spek, 2009 ▶); software used to prepare material for publication: *SHELXL97*.

## Supplementary Material

Crystal structure: contains datablocks global, I. DOI: 10.1107/S1600536809037301/ng2642sup1.cif

Structure factors: contains datablocks I. DOI: 10.1107/S1600536809037301/ng2642Isup2.hkl

Additional supplementary materials:  crystallographic information; 3D view; checkCIF report

## Figures and Tables

**Table 1:** Hydrogen-bond geometry (Å, °)

*D*—H⋯*A*	*D*—H	H⋯*A*	*D*⋯*A*	*D*—H⋯*A*
C7—H7*B*⋯S2^i^	0.97	3.00	3.793 (6)	140

Symmetry code: (i) 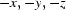.

**Fig. 1.**
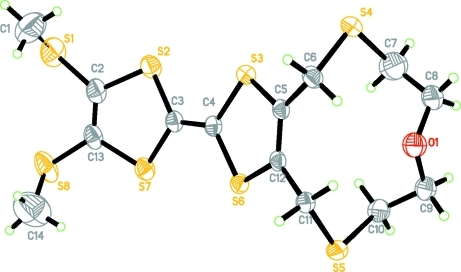
The asymmetric of title compound, with the atom numbering. Displacement ellipsoids of non-H atoms are drawn at the 30% probalility level.

## supplementary crystallographic information

### Comment

Tetrathiafulvalene (TTF) derivatives with a fused crown ether ring have received much attention as component molecules for cation sensors (Hansen *et al.*, 1993). We are incorporated TTF with a sulfur hybrid crown ether to synthesize the title compound

The molecule structure of tiltle compound, (I), C_14_H_18_S_8_O, as shown in Fig. 1, all bond lengths and angles are normal and comparable with the related structure (Hou *et al.*, 2009). In the crystal, weak intermolecular C—H···S hydrogen bonds (Table 1) link the molecules into dimer.

### Experimental

The title compound, (I), was prepared according to literature (Chen *et al.*,2005) and the single crystals suitable for X-ray diffraction were prepared by slow evaporation a mixture of dichloromethane and petroleum (60–90 °C) at room temperature.

### Refinement

Carbon-bound H-atoms were placed in calculated positions (C—H 0.93 to 0.97 Å) and were included in the refinement in the riding model with *U*_iso_(H) = 1.2 or 1.5 *U*_eq_(C).

### Crystal data

**Table:**

C_14_H_18_OS_8_	*Z* = 2
*M**_r_* = 458.76	*F*(000) = 476
Triclinic, *P*1	*D*_x_ = 1.510 Mg m^−^^3^
Hall symbol: -P 1	Mo *K*α radiation, λ = 0.71073 Å
*a* = 8.4542 (17) Å	Cell parameters from 8201 reflections
*b* = 10.158 (2) Å	θ = 3.1–27.5°
*c* = 13.612 (3) Å	µ = 0.88 mm^−^^1^
α = 105.00 (3)°	*T* = 291 K
β = 97.83 (3)°	Block, yellow
γ = 112.22 (3)°	0.14 × 0.12 × 0.12 mm
*V* = 1008.8 (3) Å^3^	

### Data collection

**Table:**

Rigaku R-AXIS RAPID diffractometer	4572 independent reflections
Radiation source: fine-focus sealed tube	3655 reflections with *I* > 2σ(*I*)
graphite	*R*_int_ = 0.032
ω scans	θ_max_ = 27.5°, θ_min_ = 3.1°
Absorption correction: multi-scan (*ABSCOR*; Higashi, 1995)	*h* = −10→10
*T*_min_ = 0.886, *T*_max_ = 0.901	*k* = −11→13
9961 measured reflections	*l* = −17→17

### Refinement

**Table:**

Refinement on *F*^2^	Primary atom site location: structure-invariant direct methods
Least-squares matrix: full	Secondary atom site location: difference Fourier map
*R*[*F*^2^ > 2σ(*F*^2^)] = 0.056	Hydrogen site location: inferred from neighbouring sites
*wR*(*F*^2^) = 0.170	H-atom parameters constrained
*S* = 1.10	*w* = 1/[σ^2^(*F*_o_^2^) + (0.0884*P*)^2^ + 0.6622*P*] where *P* = (*F*_o_^2^ + 2*F*_c_^2^)/3
4572 reflections	(Δ/σ)_max_ = 0.016
210 parameters	Δρ_max_ = 1.09 e Å^−^^3^
18 restraints	Δρ_min_ = −0.64 e Å^−^^3^

### Special details

**Table:**

Experimental. (See detailed section in the paper)
Geometry. All e.s.d.'s (except the e.s.d. in the dihedral angle between two l.s. planes) are estimated using the full covariance matrix. The cell e.s.d.'s are taken into account individually in the estimation of e.s.d.'s in distances, angles and torsion angles; correlations between e.s.d.'s in cell parameters are only used when they are defined by crystal symmetry. An approximate (isotropic) treatment of cell e.s.d.'s is used for estimating e.s.d.'s involving l.s. planes.
Refinement. Refinement of *F*^2^ against ALL reflections. The weighted *R*-factor *wR* and goodness of fit *S* are based on *F*^2^, conventional *R*-factors *R* are based on *F*, with *F* set to zero for negative *F*^2^. The threshold expression of *F*^2^ > σ(*F*^2^) is used only for calculating *R*-factors(gt) *etc*. and is not relevant to the choice of reflections for refinement. *R*-factors based on *F*^2^ are statistically about twice as large as those based on *F*, and *R*- factors based on ALL data will be even larger.

### Fractional atomic coordinates and isotropic or equivalent isotropic displacement parameters (Å^2^)

**Table:**

	*x*	*y*	*z*	*U*_iso_*/*U*_eq_	
C1	0.0932 (11)	0.6629 (10)	0.3460 (6)	0.147 (3)	
H1A	0.1923	0.7484	0.3443	0.220*	
H1B	0.0094	0.6954	0.3720	0.220*	
H1C	0.0378	0.5904	0.2761	0.220*	
C2	0.3190 (6)	0.5333 (5)	0.3702 (3)	0.0698 (11)	
C3	0.4341 (5)	0.4102 (4)	0.2243 (3)	0.0507 (7)	
C4	0.4512 (4)	0.3545 (3)	0.1275 (2)	0.0461 (7)	
C5	0.3940 (4)	0.2608 (3)	−0.0741 (2)	0.0413 (6)	
C6	0.2894 (5)	0.2050 (4)	−0.1861 (3)	0.0525 (8)	
H6A	0.3691	0.2087	−0.2314	0.063*	
H6B	0.2366	0.2724	−0.1949	0.063*	
C7	0.2424 (8)	−0.0950 (6)	−0.2145 (6)	0.1025 (17)	
H7A	0.3145	−0.0519	−0.1423	0.123*	
H7B	0.1583	−0.1969	−0.2240	0.123*	
C8	0.3534 (6)	−0.1067 (5)	−0.2800 (5)	0.0943 (17)	
H8A	0.2905	−0.1288	−0.3517	0.113*	
H8B	0.3794	−0.1915	−0.2786	0.113*	
C9	0.6658 (6)	0.0075 (5)	−0.2091 (4)	0.0740 (12)	
H9A	0.7526	0.0358	−0.2487	0.089*	
H9B	0.6321	−0.0978	−0.2166	0.089*	
C10	0.7491 (5)	0.1017 (4)	−0.0953 (3)	0.0649 (10)	
H10A	0.8265	0.0635	−0.0660	0.078*	
H10B	0.6563	0.0882	−0.0589	0.078*	
C11	0.7040 (4)	0.3605 (4)	−0.0996 (3)	0.0469 (7)	
H11A	0.7589	0.4682	−0.0874	0.056*	
H11B	0.6438	0.3094	−0.1740	0.056*	
C12	0.5700 (4)	0.3284 (3)	−0.0371 (2)	0.0407 (6)	
C13	0.4928 (6)	0.6013 (4)	0.4070 (3)	0.0661 (10)	
C14	0.7619 (13)	0.8815 (12)	0.4955 (7)	0.176 (4)	
H14A	0.8239	0.8368	0.4545	0.264*	
H14B	0.8442	0.9598	0.5587	0.264*	
H14C	0.7053	0.9239	0.4552	0.264*	
O1	0.5140 (4)	0.0229 (3)	−0.2531 (2)	0.0707 (7)	
S1	0.1683 (2)	0.5780 (2)	0.43099 (13)	0.1162 (6)	
S2	0.22953 (15)	0.37585 (11)	0.25246 (8)	0.0674 (3)	
S3	0.26931 (11)	0.23691 (10)	0.01898 (7)	0.0513 (2)	
S4	0.11564 (12)	0.01405 (12)	−0.22817 (9)	0.0704 (3)	
S5	0.87487 (12)	0.30094 (10)	−0.06741 (8)	0.0602 (3)	
S6	0.65644 (11)	0.38520 (10)	0.10015 (6)	0.0504 (2)	
S7	0.61409 (14)	0.52278 (11)	0.33593 (7)	0.0619 (3)	
S8	0.6096 (2)	0.74971 (15)	0.52682 (9)	0.1030 (5)	

### Atomic displacement parameters (Å^2^)

**Table:**

	*U*^11^	*U*^22^	*U*^33^	*U*^12^	*U*^13^	*U*^23^
C1	0.123 (4)	0.173 (5)	0.156 (5)	0.087 (4)	0.053 (4)	0.031 (4)
C2	0.087 (3)	0.059 (2)	0.060 (2)	0.022 (2)	0.044 (2)	0.0171 (18)
C3	0.062 (2)	0.0419 (16)	0.0491 (17)	0.0220 (15)	0.0185 (14)	0.0151 (13)
C4	0.0529 (18)	0.0395 (15)	0.0474 (16)	0.0223 (13)	0.0130 (13)	0.0134 (13)
C5	0.0474 (16)	0.0328 (13)	0.0428 (15)	0.0202 (12)	0.0075 (12)	0.0091 (11)
C6	0.0540 (19)	0.0458 (17)	0.0492 (17)	0.0240 (15)	−0.0016 (14)	0.0069 (14)
C7	0.090 (3)	0.068 (3)	0.143 (4)	0.032 (2)	0.033 (3)	0.029 (3)
C8	0.068 (3)	0.057 (2)	0.122 (4)	0.027 (2)	0.009 (3)	−0.017 (3)
C9	0.065 (2)	0.053 (2)	0.098 (3)	0.0339 (19)	0.016 (2)	0.005 (2)
C10	0.063 (2)	0.0484 (19)	0.089 (3)	0.0315 (18)	0.0179 (19)	0.0217 (19)
C11	0.0480 (17)	0.0389 (15)	0.0520 (17)	0.0195 (13)	0.0122 (13)	0.0119 (13)
C12	0.0467 (16)	0.0331 (13)	0.0423 (15)	0.0210 (12)	0.0087 (12)	0.0083 (11)
C13	0.089 (3)	0.0504 (19)	0.0500 (19)	0.019 (2)	0.0335 (19)	0.0126 (16)
C14	0.172 (5)	0.168 (5)	0.142 (5)	0.025 (4)	0.065 (4)	0.040 (4)
O1	0.0666 (17)	0.0519 (15)	0.090 (2)	0.0287 (13)	0.0162 (14)	0.0151 (14)
S1	0.1153 (12)	0.1136 (11)	0.1043 (11)	0.0336 (9)	0.0798 (10)	0.0109 (9)
S2	0.0694 (6)	0.0561 (5)	0.0642 (6)	0.0132 (5)	0.0322 (5)	0.0144 (4)
S3	0.0450 (4)	0.0472 (4)	0.0537 (5)	0.0173 (3)	0.0116 (3)	0.0093 (3)
S4	0.0446 (5)	0.0586 (6)	0.0766 (7)	0.0161 (4)	0.0007 (4)	−0.0092 (5)
S5	0.0433 (5)	0.0514 (5)	0.0791 (6)	0.0218 (4)	0.0133 (4)	0.0108 (4)
S6	0.0480 (4)	0.0542 (5)	0.0435 (4)	0.0262 (4)	0.0036 (3)	0.0059 (3)
S7	0.0723 (6)	0.0586 (5)	0.0457 (5)	0.0226 (5)	0.0151 (4)	0.0123 (4)
S8	0.1440 (13)	0.0685 (7)	0.0543 (6)	0.0108 (8)	0.0411 (7)	0.0005 (5)

### Geometric parameters (Å, °)

**Table:**

C1—S1	1.794 (9)	C8—O1	1.405 (6)
C1—H1A	0.9600	C8—H8A	0.9700
C1—H1B	0.9600	C8—H8B	0.9700
C1—H1C	0.9600	C9—O1	1.421 (5)
C2—C13	1.319 (7)	C9—C10	1.495 (6)
C2—S1	1.744 (4)	C9—H9A	0.9700
C2—S2	1.768 (4)	C9—H9B	0.9700
C3—C4	1.344 (5)	C10—S5	1.798 (4)
C3—S2	1.747 (4)	C10—H10A	0.9700
C3—S7	1.756 (4)	C10—H10B	0.9700
C4—S3	1.747 (3)	C11—C12	1.498 (4)
C4—S6	1.753 (3)	C11—S5	1.809 (3)
C5—C12	1.335 (4)	C11—H11A	0.9700
C5—C6	1.496 (4)	C11—H11B	0.9700
C5—S3	1.762 (3)	C12—S6	1.763 (3)
C6—S4	1.814 (4)	C13—S8	1.752 (4)
C6—H6A	0.9700	C13—S7	1.765 (4)
C6—H6B	0.9700	C14—S8	1.665 (9)
C7—C8	1.397 (8)	C14—H14A	0.9600
C7—S4	1.833 (6)	C14—H14B	0.9600
C7—H7A	0.9700	C14—H14C	0.9600
C7—H7B	0.9700		
			
S1—C1—H1A	109.5	C10—C9—H9A	108.9
S1—C1—H1B	109.5	O1—C9—H9B	108.9
H1A—C1—H1B	109.5	C10—C9—H9B	108.9
S1—C1—H1C	109.5	H9A—C9—H9B	107.7
H1A—C1—H1C	109.5	C9—C10—S5	116.1 (3)
H1B—C1—H1C	109.5	C9—C10—H10A	108.3
C13—C2—S1	125.9 (3)	S5—C10—H10A	108.3
C13—C2—S2	117.2 (3)	C9—C10—H10B	108.3
S1—C2—S2	116.8 (3)	S5—C10—H10B	108.3
C4—C3—S2	123.3 (3)	H10A—C10—H10B	107.4
C4—C3—S7	123.7 (3)	C12—C11—S5	113.4 (2)
S2—C3—S7	112.98 (18)	C12—C11—H11A	108.9
C3—C4—S3	122.6 (3)	S5—C11—H11A	108.9
C3—C4—S6	123.2 (3)	C12—C11—H11B	108.9
S3—C4—S6	114.08 (18)	S5—C11—H11B	108.9
C12—C5—C6	127.3 (3)	H11A—C11—H11B	107.7
C12—C5—S3	116.9 (2)	C5—C12—C11	127.1 (3)
C6—C5—S3	115.8 (2)	C5—C12—S6	117.2 (2)
C5—C6—S4	113.5 (3)	C11—C12—S6	115.7 (2)
C5—C6—H6A	108.9	C2—C13—S8	125.2 (3)
S4—C6—H6A	108.9	C2—C13—S7	116.7 (3)
C5—C6—H6B	108.9	S8—C13—S7	117.7 (3)
S4—C6—H6B	108.9	S8—C14—H14A	109.5
H6A—C6—H6B	107.7	S8—C14—H14B	109.5
C8—C7—S4	119.7 (5)	H14A—C14—H14B	109.5
C8—C7—H7A	107.4	S8—C14—H14C	109.5
S4—C7—H7A	107.4	H14A—C14—H14C	109.5
C8—C7—H7B	107.4	H14B—C14—H14C	109.5
S4—C7—H7B	107.4	C8—O1—C9	114.7 (4)
H7A—C7—H7B	106.9	C2—S1—C1	100.8 (3)
C7—C8—O1	114.6 (4)	C3—S2—C2	94.04 (19)
C7—C8—H8A	108.6	C4—S3—C5	94.20 (15)
O1—C8—H8A	108.6	C6—S4—C7	102.0 (2)
C7—C8—H8B	108.6	C10—S5—C11	102.29 (17)
O1—C8—H8B	108.6	C4—S6—C12	93.92 (15)
H8A—C8—H8B	107.6	C3—S7—C13	94.02 (19)
O1—C9—C10	113.4 (3)	C14—S8—C13	104.6 (3)
O1—C9—H9A	108.9		

### Hydrogen-bond geometry (Å, °)

**Table:**

*D*—H···*A*	*D*—H	H···*A*	*D*···*A*	*D*—H···*A*
C7—H7B···S2^i^	0.97	3.00	3.793 (6)	140

Symmetry codes: (i) −*x*, −*y*, −*z*.
